# Assessing Digital Transformation of Cost Accounting Tools in Healthcare

**DOI:** 10.3390/ijerph192315572

**Published:** 2022-11-23

**Authors:** Anca Antoaneta Vărzaru

**Affiliations:** Department of Economics, Accounting and International Business, University of Craiova, 200585 Craiova, Romania; anca.varzaru@edu.ucv.ro

**Keywords:** digital transformation accounting management, cost accounting tools, users’ perception, healthcare

## Abstract

The expansion of digital technologies has significantly changed most economic activities and professions. Digital technologies penetrated managerial accounting and have a vast potential to transform this profession. Implementing emerging digital technologies, such as artificial intelligence, blockchain, the Internet of Things, big data, and cloud computing, can trigger a crucial leap forward, leading to a paradigm-shifting in healthcare organizations’ accounting management. The paper’s main objective is to investigate the perception of Romanian accountants on implementing digital technologies in healthcare organizations’ accounting management. The paper implies a study based on a questionnaire among Romanian accountants who use various digital technologies implemented in traditional and innovative cost accounting tools. Based on structural equation modeling, the results emphasize the prevalence of innovative tools over traditional cost accounting tools improved through digital transformation, digital technologies assuming the most complex and time-consuming tasks. Moreover, the influence of cost accounting tools improved through digital transformation on healthcare organizations’ performance is much more robust in the case of innovative tools than in the case of traditional cost accounting tools. The proposed model provides managers in healthcare organizations with information on the most effective methods in the context of digital transformation.

## 1. Introduction

Over the last decade, technological advances generated by the increasing use of technologies, such as artificial intelligence (AI), big data (BD), blockchain (BC), cloud computing (CC), and the Internet of Things (IoT), were the vectors of the technological revolution known as Industry 4.0. The emergent technologies significantly affect cost reduction, efficiency enhancement, and profit-boosting [1]. Various industries and service activities [2], including the healthcare industry [3] and public administration [4], implemented these emerging technologies in their early stages. Research and reports of professional bodies highlighted the significant changes already applied and those that will take place in the future in accounting management (AM) [5,6,7,8]. Digital transformation (DT) in AM began several decades ago with the implementation of IT solutions. Nevertheless, the entire process of AM is undergoing profound and paradigmatic transformation with the implementation of AI, BD, BC, CC, or IoT technologies.

New technologies make it possible to process and interpret large amounts of data in real time, strengthening transparency and generating increased confidence. Various researchers [9,10,11,12,13,14,15,16,17,18] show that IoT, BD, AI, and CC used in healthcare improved planning, decision-making, and treatment with a reduced time and cost. IoT ensures decision-making in time [10,11,12,13,14,18], based on the collection and processing of BD [9,15,17], shared with CC technology [10,16]. AI solves routing, traffic engineering, resource allocation, and security [10,15]. Combined, new digital technologies could enhance effective healthcare delivery [10,15,17]. New technologies are helpful throughout the AM process, from data collection to final decision-making. New technologies can facilitate and efficiently perform most operations within cost accounting tools (CAT), both traditional and innovative in the healthcare industry. (AI, BD, BC, CC, or IoT). IT solutions for costing, such as EasyKost, CostPerform, Boothroyd Dewhurst DFMA Software, Price Cost Analytics (PCA), or IT solutions for managing and planning enterprise resources, such as Oracle NetSuite, SAP ERP, Acumatica, and BizAutomation, are software that has dramatically transformed cost accounting. The next step, however, is the integration of technologies, such as AI, BD, BC, CC, or IoT within these IT solutions to make cost accounting more efficient. Some IT solutions, such as Oracle ERP Cloud and SAP S/4 HANA, already integrate AI, CC, or IoT technologies. However, user acceptance of digital technologies in already existing IT solutions is a gap that the paper identified in the digital accounting literature. The complexity of the activities, the multitude of cost elements, the specifics of the clients (patients who benefit from medical services), the characteristics regarding the privacy of the medical act and the transparency of expenses with medical services, and the difficulty of evaluating the activities of physicians and auxiliary staff represent specific features of accounting management in the healthcare industry.

Based on the gap found in the literature, this study aims to evaluate the effects of DT generated by implementing emerging technologies, such as AI, BD, BC, CC, or IoT, in the traditional and innovative CAT. The paper highlights the impact of the costing activity digitalization on improving costing processes and organizational performance in the healthcare industry. The empirical study implies a survey based on a questionnaire given to accountants with seniority in cost accounting in the Romanian healthcare industry. The paradigm-shifting in costing activity due to the DT was investigated by reviewing the literature. The study has an innovative character through the research topic addressed (DT of costing tools) and its practical utility for specialists in AM in the healthcare industry. Most costing operations can be translated into a controlled and accessible digital environment.

The structure of the paper has six sections: the introduction is followed by the literature review, methodology, and findings sections. Finally, the paper ends with discussions, conclusions, research, and future research directions.

## 2. Literature Review

The first step was the systematic literature review on traditional and innovative CAT and how these existing IT solutions can be improved through DT using emerging technologies, such as AI, BD, BC, CC, and IoT. DT can increase organizational performance through more effective accounting management. DT in all areas, including AM, increased exponentially, particularly after the COVID-19 pandemic due to mobility restrictions [6].

### 2.1. Cost Accounting Tools

Among the traditional CAT methods most used today are still standard costing (SC), absorption costing (AC), process costing (PC), and marginal costing (MC) [19]. The most widely used innovative CAT in costing activities within companies are activity-based costing (ABC), target costing (TC), life cycle costing (LC), and Kaizen costing (KC) [19]. Although many researchers claim that traditional CAT have lost their relevance and should no longer be used in accounting management [20,21,22], there are many traditional tools that, although strongly criticized, are still used extensively in developing countries. The choice of CAT depends on the contextual analysis, i.e., the company’s size, the national and organizational culture, human resources skills, and, lately, technological advancement.

Table 1 shows the most common traditional CAT used in organizations’ accounting management. 

In the last three decades, several researchers have criticized traditional CAT, claiming that costing activity in AM has lost its relevance due to innovations and the dynamism of business environments [19,28,29]. Criticisms of the reduction in the relevance of traditional CAT have led to the emergence of new CAT in cost accounting, such as ABC, TC, LC, or KC. Although new CAT have been widely introduced in cost calculation, traditional CAT are still widely used. Table 2 shows the most common innovative CAT used in the organizations’ AM.

Dugdale et al. [43] show that traditional managerial accounting tools are still widely used alongside innovative tools. Old tools are still helpful in some contexts, taught in business schools, and used in practice, sometimes alone in combination with other traditional or innovative costing tools. Bhimani and Willcocks [44] and Bhimani [35] show that, although innovative tools have been introduced in many companies, traditional tools are still used. DT generated by implementing technologies, such as AI, BD, BC, CC, or IoT, in existing IT solutions facilitates using all traditional and innovative tools, offering high data collection capacity, access to real-time data, high computing power, interpretation capabilities, and decision-making.

The paper proposes the first hypothesis regarding CAT based on the analysis of the literature and findings on the costing activity in the Romanian healthcare industry:

**Hypothesis** **H1.** *In the users’ perception, the most valuable traditional CAT is AC, while the most useful innovative CAT is TC.*

### 2.2. Digital Technologies Used in Costing

AI technology reduces the time allocated by accountants to intermediate processing by allowing them to engage in higher-value activities: decision-making, monitoring costing processes, and updating or modifying AI costing tools improving performance in all AM activities and contributing to increased transparency [6,45,46]. In addition, AI facilitates the analysis and interpretation of accounting information, enabling price management decisions [15,47]. In the field of costing, AI is also used to estimate target costs, using analogical and parametric cost estimation methods based on historical costs. Recent advances in the development of algorithms and machine learning processes improved traditional methods, bringing into question the nonparametric approach (e.g., the Random Forests algorithm, proposed by [48] and [49], implies multiple decision trees to improve predictive performance).

BD technology offers several significant advantages for costing tools, particularly in the healthcare industry, given the disadvantages related to the large amounts of information and the complexity of the data collected. In addition to traditional information in a quantifiable form, BD can enhance information using data, images, audio data, and data generated by IoT sensors, including patients [6,15,17]. These data are in addition to existing records, providing an opportunity to improve AM practices.

BC and IoT can directly impact accounting records; the BC is a distributed registry based on data decentralization and ensuring transparency and security through cryptography [50,51,52]. Data collected by accountants or directly through IoT technology is saved on a block of records linked to previous blocks using cryptography. The cost data recorded can be checked at any time [50]. The essential feature of the BC that makes it important for costing tools is the impossibility of changing records: these changes can only be made after obtaining network acceptance [51]. Therefore, modifying, alternating, and manipulating data is extremely difficult, generating a high trust in that data. BC, integrating AI and BD functions, will allow data collection, processing, fast sharing, and automatic control, significantly contributing to the development of new costing systems.

BC technology eliminates the need to replicate information in multiple databases [53], accelerating information sharing with CC technology and significantly reducing human error, an essential aspect of costing activity. In addition, decentralization, immutability, verification, and traceability make the information used in the costing activity more reliable [54].

IoT makes it possible to obtain data based on sensors embedded in various assets or positioned in the workplace, including in the patients’ rooms, transforming the physical world into an information system [10,11,12,13,14,55]. Built-in sensors transmit real-time cost data, which accountants can retrieve directly or transmit in a cloud. The data collected allow for a correct assessment of costs in the healthcare industry, providing real-time measurements of various costs [56]. The actual costs determined based on the data transmitted by the sensors will replace the arbitrary allocations [57]. CC and BC systems allow information to be shared directly between network components, eliminating the need for an intermediary [58]. The data collected using several sensors, considered objective sources, can offer accountants in the healthcare industry a more accurate and integrated view of costs and constitute the basis of the right decisions. IoT will increase the reliability of costing activities through the four characteristics of the technology: access to real-time data, interoperability, digitization of information, and decentralization [55,59,60].

CC involves storing and managing large and complex amounts of data and sharing them with all stakeholders, including patients and relatives [10,61,62,63]. In this way, some operations of the costing systems are automated. In addition, CC platforms are constantly improving system security, eliminating security and privacy concerns, which is essential in healthcare. [64,65]. CC enables accountants to carry out their activities effectively, ensuring data security and synchronization and reducing the risk of asynchronous data. Therefore, CC will be essential in automated CAT, helping collect and produce information concerning costs in the healthcare industry.

Many researchers present the advantages of using CC technology in AM of the healthcare industry. For example, according to [6,10,16,66,67,68], the application of CC technologies in the costing activity allows the simplification of accounting documents, the translation of operations in the information environment based on cloud networks, the cost savings generated by information transmission, improving the quality of the decision-making process through transparency and increasing access to information, increasing the possibilities for communication between accountants, and eliminating the risks of data desynchronization.

The accounting data required for costing tools can be collected and made available to various categories of users using CC systems. Decision-making in costing tools uses large volumes of data (BD) and AI to increase the rapidity of decisions, eliminate human error and reduce the time and costs with human resources involved [69]. Yoon [6] points out that robotic process automation and machine learning can be used to obtain more significant and relevant cost information, which can lead to better-informed decisions. The use of BC technology increases the quality of organizational cost information and ensures transparency, reliability, and real-time access to this information.

The paper formulated three hypotheses regarding the essential characteristics of DT implementation in AM, the effects of DT implementation in AM, and the effects on organizational performance based on the analysis of the literature on DT and our findings:

**Hypothesis** **H2.** *Among the DT antecedents, the most important determinants are rapidity and customization in the users’ perception.*

**Hypothesis** **H3.** *In the users’ perception, the most significant positive influence of the DT implementation is exerted on innovative CAT.*

**Hypothesis** **H4.** *Innovative CAT improved through DT exerts a significant favorable influence on organizational performance in the users’ perception.*

## 3. Methodology

### 3.1. Research Design

To study the impact of DT on traditional and innovative CAT and organizational performance, the paper investigates perceptions of Romanian accountants with seniority in cost accounting in the healthcare industry

The most critical aspect of DT in CAT is the acceptance of implementing digital technologies, such as AI, BD, BC, CC, or IoT, within existing IT solutions and their actual use due to the perception of increased efficiency and effectiveness of the activity. Even if healthcare organizations invest large amounts of money in implementing digital technologies, it is necessary to understand the acceptance by users of the new technologies [70]. Starting from the technology acceptance model (TAM) proposed by Davis [71], various studies applied the TAM model regarding new technology acceptance in various fields [72,73,74,75,76,77,78]. Following a meta-analysis of the specialized literature, King and He [75] demonstrated that perceived usefulness is the most important predictor of behavioral intention. Considering the specifics of the use (for professional purposes) of the new technologies, we consider that the perceived usefulness has a determining role in technology acceptance, as shown [75].

### 3.2. Selected Variables

**Figure 1:**
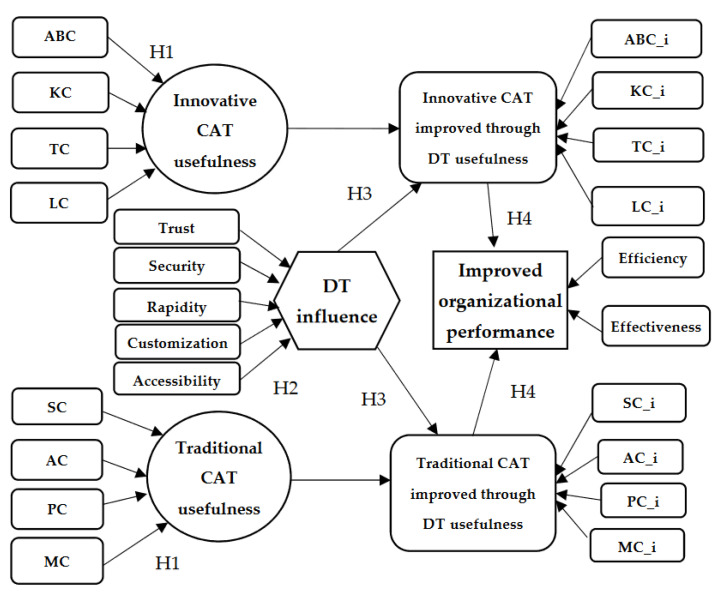
Theoretical model. Source: own construction.

The research design considered four traditional CAT (SC, AC, PC, and MC) and four innovative CAT (ABC, TC, LC, and KC). The perception of Romanian accountants in the healthcare industry regarding the usefulness of CAT represents exogenous variables of the theoretical research model, which determine the latent variables of innovative CAT usefulness and traditional CAT usefulness. The latent variable DT influence has antecedents defined based on the literature in the field: trust, security, rapidity, customization, and accessibility [70,71,72,73,74,75]. The extent to which the application of CAT could be improved by implementing DT, in the perception of Romanian accountants in the healthcare industry, defines two other latent variables which have as antecedents the four traditional CAT (SC, AC, PC, and MC) and the four innovative CAT (ABC, TC, LC, and KC) improved through DT. The fifth variable, organizational performance, has antecedents in the efficiency and effectiveness of organizational activities. Figure 1 illustrates the theoretical research model. 

### 3.3. Selected Sample and Methods

Romanian accountants’ perceptions concerning DT influence on traditional and innovative CAT and organizational performance were collected using a questionnaire to test the theoretical model and the four hypotheses. The questionnaire comprises items of the exogenous variables of the theoretical model (Table A1). The questionnaire was filled out online by 423 accountants from Romanian organizations in the healthcare industry between March 2022 and May 2022. Five hundred eighty-six questionnaires were sent, 438 questionnaires were returned, of which 423 were filled, and the participation rate was 72.18%. The sampling method was stratified randomly, depending on the criteria of gender and age. The respondents provide answers voluntarily and anonymously, contributing to combating common biases. The questionnaire includes general questions regarding perceptions of the use of new technologies and does not include data that requires an institutional review board and informed consent statement. The items used a Likert scale with five levels for the DT, CAT, and organizational performance variables. Table 3 illustrates the structure of the questionnaire and the scales used.

The use of self-administered questionnaires can generate the problem of common method bias [79]. Using Harman’s single-factor test, the paper tested all variables by factor analysis using principal component analysis. As a result, the total variance extracted was below 50% (46.834%), proving no substantial bias effects [79]

The paper uses structural equation modeling (SEM) to test the theoretical model and determine the influences within the theoretical model. The formula used is [80]:

(1) $$\eta_{i}=\alpha_{\eta }+B\eta_{i}+\Gamma \xi_{i}+\zeta_{i}$$

η,ξ—endogenous and exogenous latent variables vectors, 

Β—matrix of regression coefficients relating the latent endogenous variables to each other, 

Γ—matrix of regression coefficients relating the endogenous variables to exogenous variables, 

ζ—disturbance,

i—cases in the sample.

The latent variables are related to exogenous variables as follows:

(2) $$y=\Lambda_{y}\eta +\varepsilon ,$$

(3) $$x=\Lambda_{x}\xi +\delta ,$$

where:

*Λ_y_*, *Λ_x_*—matrices of factor loadings;

*ε*, *δ***—**vectors of uniqueness.

## 4. Results

**Figure 2:**
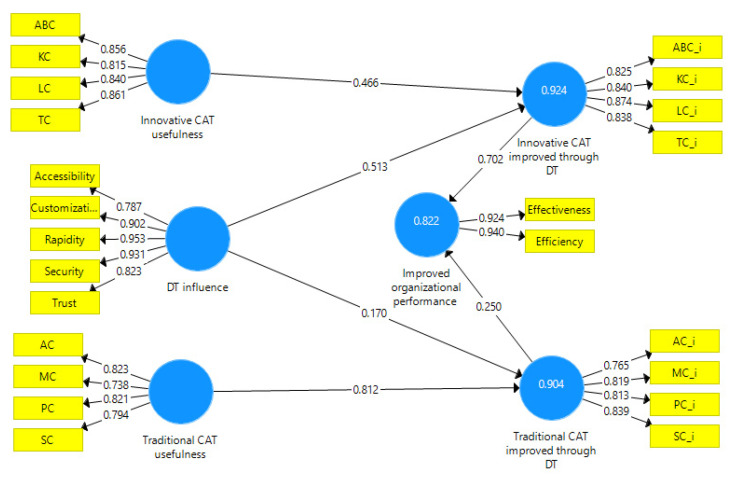
Empirical model. Source: own construction using SmartPLS v3.0 (SmartPLS GmbH, Oststeinbek, Germany).

To investigate the influence of DT on traditional and innovative CAT and organizational performance proposed in the theoretical model, the paper use SmartPLS v3.0 (SmartPLS GmbH, Oststeinbek, Germany), which conducted SEM in a partial least square variant. The investigation uses a reflective model. PLS algorithm is used for validation, outer loadings, and outer weights, while a bootstrapping procedure supplies path coefficients in SmartPLS 3.0. Figure 2 shows the theoretical model applied.

The reliability and validity are excellent (Table 4). Also, the model records values below 0.08 (0.078) for SRMR (standardized root mean squared residual) and values over 0.9 (0.917) for NFI (normed fit index), proving a good fit for the model.

All validity and reliability measures presented in Table 4 confirm good reliability and validity, according to [80].

Confirmation of the validity of hypotheses H1 and H2 involved the analysis of outer loadings and outer weights to determine the most important antecedent of the latent variables. (Table 5).

The analysis of the outer loadings and outer weights in Table 4 confirms that hypothesis H1 is valid. The most useful traditional CAT in the users’ perception is AC, while the most useful innovative CAT in the users’ perception is TC. The results are similar to other research conducted among accountants in other countries [9,26,71].

Among the antecedents of the DT influence, the most important are rapidity and security in the users’ perception, which confirms a partial validation of the H2 hypothesis. The real-time feature of CAT operations makes rapidity the antecedent with the highest outer loading and outer weights. Other research results confirm the importance of real-time accounting operations [55,59,60]. Although data security is a crucial issue, especially in financial accounting and auditing, the users indicated information security as a critical feature of digital technologies, including cost accounting in the healthcare industry.

Following a bootstrapping procedure in SmartPLS 3.0 (with 500 subsamples and a significance level of 0.05), the path coefficients indicate significant positive direct influences among model variables (Table 6). Values above 1.6 for T statistics and below 0.005 for *p* values show an increased relevance of the path coefficients [70].

Path coefficients in Table 5 reveal a more substantial positive influence of digital technologies on the innovative CAT than in the case of the traditional CAT, which confirms the H3 hypothesis. Furthermore, the validity of the H4 hypothesis confirms the significant positive influence of the perceived innovative CAT improved through DT usefulness on organizational performance, compared to the perceived traditional CAT improved through DT usefulness, in line with the results of other research [9,10,11,12,13,14,15,16].

## 5. Discussion

The expansion of Industry 4.0 has led to the development of most cost accounting tools, both traditional and innovative CAT, including in the healthcare industry. The development of cost accounting and control practices in modern companies took place mainly between 1850 and 1925 [28] when most traditional CAT emerged, with the rise of scientific management. The information provided by traditional CAT helped analyze operational efficiency in the decision-making process regarding pricing and the activities regarding control and motivation. The main disadvantage of the traditional CAT is a limited vision at the workplace level or the cost unit and an incomplete overview of the financial and general performance [22]. The paper analyzed the perception of selected respondents (Romanian accountants in the healthcare industry) on traditional and innovative CAT. Similar to other research [19,26,81], the paper found the prevalence of innovative CAT over traditional CAT, but traditional CAT are still intensely used in the healthcare industry due to lower costs, time consumption, and lack of skills. The investigation of the H1 hypothesis revealed that absorption costing is the most used tool in the healthcare industry among the traditional CAT. In contrast, among the innovative CAT, the most used tool in the healthcare industry is target costing, based on outer loading and outer weights of exogenous variables. The results were in line with the findings of other researchers [28,29,30].

The digitization of cost accounting in the healthcare industry supposes the automation of the accounting processes to facilitate the accomplishment of routine tasks and access to information in real-time on costs [9,10,11,12,13,14,15,16,17,18,82,83]. In line with the findings of other authors [81,82,83,84], the paper on the H2 hypothesis revealed the characteristics of the digital technologies implemented in traditional and innovative CAT appreciated by the respondents. The rapidity of operations (in real-time) and information security are the essential features of digital technologies implemented in traditional and innovative CAT that influence their efficiency and effectiveness. Moreover, the other features selected from the literature on technology acceptance [54,55,59,60,61,62,63,70,78], i.e., customization, trust, and accessibility, record high values of outer loadings and outer weights in the healthcare industry, proving their relevance for accountants.

Currently, the entire AM process undergoes a profound and paradigmatic DT through AI, BD, BC, CC, or IoT technologies [6]. While CC makes it possible to store and use BD, BD and IoT can be combined with AI technology [10,56]. BD is a critical component of AI technology because AI is based on machine learning. Through CC, data on costs collected and IoT can be stored, and AI can effectively process and interpret these BD, helping substantiate cost decisions. Therefore, new digital technologies underlie the construction of more advanced costing IT solutions, providing high-quality information with substantial time and cost savings and contributing to accounting transparency and autonomy in the healthcare industry.

Regarding information transparency and reliability, BC technology can play a crucial role in contributing to information security activities. Manipulating cost records is extremely difficult because they are shared by all network participants using cryptography [56]. The costing accounting process can be considerably developed by joining each item of innovative technologies, as found by other researchers who applied the TAM model in other fields [72,73,74,75,76,77,78]. By validating the H3 hypothesis, the paper confirmed the significant positive influence of DT on CAT in the healthcare industry, with innovative CAT being much more useful in users’ perception after implementing digital technologies than traditional CAT.

Implementing information technologies in the costing activity brings several advantages [84]. DT allows simple and repetitive tasks in a short time and without human effort, supported by CC technology that permits quick sharing of information. BC provides high information security and improves transparency. Activities that involve a large amount of information and are time-consuming, such as costing, can be improved by automating costing processes, leading to the improved overall performance of the organization [54,63]. In line with the findings of other researchers [3,69,81], the investigation of the H4 hypothesis confirmed the influence, especially of perceived innovative CAT improved through DT usefulness on the healthcare organizations’ performance through better management of the organizational costs. The opportunities offered by digital technologies are substantial. Many processes within accounting and managerial information systems are made more efficient through innovation and digitization [9,10,11,12,13,14,15,16,17,18,85,86,87], contributing to healthcare organizations’ high performance [10,38,39,40,41,42,43,44,45,46,47,48,49,50,51,52,53,54,55,56,57,58,59,60,61,62,63,64,65,66,67,68,69]. However, there are also several risks, obstacles, and challenges. The reluctance of cost accounting professionals to adopt digital technologies in their work and use them to make their work easier and achieve better results is a significant challenge that must be addressed [88].

In addition to the advantages offered, there are several disadvantages of using these information technologies [89]: the high skills level of human resources needed to use these technologies, the need to customize for each organization which requires time and effort, and the elimination of many jobs among accountants. However, the implementation of teleworking accelerated by the COVID-19 pandemic has determined a faster acceptance of digital technologies, including in the healthcare industry [90].

Marr [91] shows that it is not difficult to use information technologies in AM because AM is not mandatory. Therefore, cost calculation offers greater freedom for implementing technologies, such as AI, BD, BC, CC, or IoT, without ensuring compliance with specific rules or standards and accounting systems, as in the case of financial accounting or audit. However, despite all the advantages offered, activities that require social intelligence cannot be performed only by AI, particularly in the healthcare industry. For example, experts are still needed to use the information provided by these digital technologies to improve estimates and predictions by taking into account social and individual factors in the cost calculation process [92]. In other words, at least at the current technological level, digital technologies can provide helpful information. However, the final decisions must belong to human beings, who can identify based on intuition and emotional intelligence factors that digital technologies ignore.

### 5.1. Theoretical Implications

CC is an essential tool that will lead to the DT of AM. CC, combined with BD and IoT, has brought various benefits to costing tools. In turn, BD is the vector underlying the use of AI technology in AM. BC can provide trust, reliability, transparency, and accessibility for costing tools that use digital technologies. DT is not just an acceptance of technologies and their formal use. The DT in cost accounting in the healthcare industry implies a paradigmatic, structural change that will lead to new costing tools in combination with new technologies. The new costing tools will derive from current IT solutions that integrate traditional or innovative tools, which will develop based on the growing capabilities offered by new technologies: AI, BD, BC, CC, or IoT.

Accountants are essential to building effective and valuable accounting and management information systems in the healthcare industry. Their collaboration with IT specialists in the design and maintenance of information systems, as creators and managers of information, can bring immense benefits to the field of accounting management [93]. The role of efficient and valuable accounting and managerial information systems in information management is essential to ensure the information necessary for decision-making in the healthcare industry [94]. New technologies transform cost accountants’ operational and tactical roles into strategic roles, accounting information systems being the basis of essential decision-making processes [95].

### 5.2. Empirical Implications

This study aims to understand how new technologies can improve the application of traditional and innovative CAT and ultimately lead to increased organizational performance. The paper conducted theoretical and empirical research on academic literature based on a questionnaire applied to accountants with seniority in cost accounting in the Romanian healthcare industry, formulating four hypotheses. The validity investigation of the hypotheses led us to conclude that among the traditional CAT, the most used technique is absorption costing in the healthcare industry, while among the innovative CAT, the most used technique is target costing. The implementation of digital technologies in the healthcare industry can lead to the improvement of both traditional and innovative CAT. Among the characteristics of digital technologies implemented in traditional and innovative CAT in the healthcare industry, the rapidity of operations (in real-time) and information security influenced CAT efficiency and effectiveness. Empirical research has demonstrated that the prevalence of innovative CAT improved through DT over traditional CAT improved through DT due to the takeover of complex and time-consuming tasks by digital technologies. Moreover, the influence of CAT improved through DT on healthcare organizations’ performance is much more robust in the case of innovative CAT than in the case of traditional CAT.

### 5.3. Limitations and Further Research

The study’s main limitation comes from limited geographical representativeness, given that the subjects of the empirical study were only Romanian accountants with seniority in cost accounting in the healthcare industry. The study focuses on their perceptions of improving costing processes through DT and the impact of this improvement on healthcare organizational performance. Another limitation comes from the transversal approach, which provides a clearer picture of the situation and does not allow the analysis of the evolution over time of accountants’ perceptions. Future research directions arise from the limits of the paper and must take into account the geographical expansion of the researched population and a longitudinal approach that allows the analysis of trends in cost accounting. Extending the research area to other organizational processes, not only the costing activity, can be an essential and integrative direction of future research in the healthcare industry. Digital transformation affects all areas of the healthcare organization, and it is essential to evaluate the acceptance of users implementing new digital technologies.

## 6. Conclusions

The costing activity will increasingly integrate into Industry 4.0 using AI, BD, BC, CC, or IoT. These technologies are emerging digital technologies with accelerated development due to the appropriate technical and social conditions. Digital technologies allow large amounts of operations based on complex volumes of data, permitting AM specialists to perform the costing activity and more grounded decision-making in the healthcare industry. Most of the respondents selected in the sample (Romanian accountants in healthcare organizations) are convinced that DT will lead to improved costing activities. At the same time, accountants are aware of the need for adaptation to the changes generated by the digitization of costing activity.

To facilitate the acceptance of new digital technologies implemented in existing IT solutions, healthcare managers must convince accountants with seniority in cost accounting that the innovations will make work more accessible and valuable, especially considering the large volume of data and the processing capabilities of the new IT solutions.

## Figures and Tables

**Table 1:** Traditional cost accounting tools.

CAT	Description	References
Standard costing	SC is a managerial accounting tool that analyzes real-cost variations compared to the standard costs pre-established in the planning phase. This costing method is used in the exception management method. Each cost element has a standard cost established scientifically (technical characteristics of the products, historical costs, etc.). The analysis of the differences between standard and actual costs makes it possible to detect deviations and take corrective measures to remove the cause of inefficiency [21,22,23,24]. Although it is a tool that allows correcting errors, it does not allow combating deviations in real time but only after the end of the production process. SC offers convenience and rapidity in calculating production costs, preparing budgets, setting product prices, and evaluating the performance of organizational divisions. However, contemporary price dynamics, rapid change in cost structure, and delayed feedback make this method ineffective, especially in high-dynamism economic sectors (such as IT).	[23,24,25,26]
Absorption costing	AC, known as the total cost method, considers all the production costs of the product or service. Whether they are variable or fixed costs, they are gradually absorbed until the total cost is reached. Financial statements use the principles of absorption costs, as this method no longer requires subsequent accounting treatments. However, AC is not very compatible with highly automated work environments without considering significant investments in IT solutions. Nevertheless, AC was relevant, considering direct labor costs were the most significant component of the total cost.	[19,20,27,28,29]
Process costing	PC is used when the organization produces homogeneous standardized goods. PC is also used in industries involving an assembly process. The calculation of costs implies accumulating all costs for each stage of production or process. Then, the unit cost is determined in each stage by dividing the cost of each process by the units produced. The process costing system is useful in industries with high innovation.	[28,30,31,32]
Marginal costing	MC has two variants: variable costing and direct costing. This costing tool assesses separately variable and fixed costs, respectively direct and indirect costs, to support the decision-making process. MC enables managers to focus on changes in cost structure and make decisions based on this information. MC is useful for supporting short-term decisions concerning purchases, sales, outsourcing a part of the production process, etc.	[26,27,28,29]

Source: own construction based on [19,20,23,24,25,26,27,28,29,30,31,32].

**Table 2:** Innovative cost accounting tools.

CAT	Description	References
Activity-based costing	ABC, designed in the mid-1980s, achieved a more appropriate and realistic allocation of the organization’s overhead. The implementation of this tool is related to combating the disadvantages of SC. ABC identifies cost factors according to the distribution of indirect expenses and the organization’s general expenses on the cost of activities. The costs of the activities are then used to form the costs of the products or services. Monitoring the activities and resource consumption allocated to each activity allows a more judicious distribution of costs by products and services. Resources are allocated to activities, and activities are allocated to cost objects based on consumption estimates. The concept of ABC formed the basis of the activity-based management (ABM) system used successfully in the services sector. ABM allows the removal of activities that do not add value and a better distribution of costs. However, ABC is expensive, time-consuming, and challenging to adjust. The DT proposed by Industry 4.0 can streamline this method.	[33,34,35,36,37]
Target costing	TC emerged in response to the challenges posed by consumer demand for diversity and the shortening of product life cycles. TC considers the costs of products in the design phase, which have become increasingly significant in a product’s total costs over its life cycle. Being a multidisciplinary approach to costing, TC involves process reengineering and total quality management techniques. The basic principle is to set a competitive price for a product, starting from the market price. Then, by subtracting a desirable profit margin, the target cost is obtained. The costing tool is suitable for the services sector and the industry, allowing a highly competitive approach. Based on the target cost, cost elements can be estimated so that the final cost fits into the target cost.	[19,27,28,38,39]
Life cycle costing	LC aims to identify the total cost associated with the entire life cycle of a product or service, considering the design costs and all the company costs related to the product. LC allows the assessment of an asset’s costs over its life cycle, making it possible to quantify the consequences of the decision and improve forecasts by understanding the trade-off between performance and cost. Disadvantages include a lack of data and high time consumption. LC is useful when launching a product requiring large initial capital outflows.	[27,28,29,35,40]
Kaizen costing	KC is based on the continuous improvement cycle involving all organization members, from top managers to simple workers. Therefore, the costing tool can increase productivity, gain a competitive advantage, and increase overall performance. In addition, KC has the advantage of minimal application costs. In addition, KC has the advantage of minimal application costs. Like ABC, this method has been associated with a management system (Kaizen Management) dedicated to improving effectiveness, efficiency, quality, and overall organizational performance. Improving the quality of processes and products increases the company’s profits and customer loyalty.	[19,41,42]

Source: own construction based on [19,27,28,29,35,36,37,38,39,40,41,42].

**Table 3:** Questionnaire structure and scales.

Variables	Items	Scales
Demographic variables	Gender	Male(1), Female (2)
	Age	18–30 years (1), 31–45 years (2), 46–65 years (3)
Innovative CAT usefulness	ABC	On a scale of 1 to 5 (1—not at all useful, 5—very useful)
	TC	
	LC	
	KC	
Traditional CAT usefulness	SC	
	AC	
	PC	
	MC	
DT influence	Trust	On a scale of 1 to 5 (1—not at all important, 5—very important)
	Security	
	Rapidity	
	Customization	
	Accessibility	
Innovative CAT improved through DT usefulness	ABC	On a scale of 1 to 5 (1—not at all useful, 5—very useful)
	TC	
	LC	
	KC	
Traditional CAT improved through DT usefulness	SC	
	AC	
	PC	
	MC	
Improved organizational performance	Efficiency	On a scale of 1 to 5 (1—very small, 5—very high)
	Effectiveness	

Source: own construction based on [67,69,70,71,72].

**Table 4:** Validity and reliability.

	Cronbach’s Alpha	Composite Reliability	AVE
DT influence	0.927	0.945	0.777
Improved organizational performance	0.849	0.930	0.868
Innovative CAT improved through DT	0.866	0.909	0.713
Innovative CAT usefulness	0.864	0.908	0.711
Traditional CAT improved through DT	0.824	0.883	0.655
Traditional CAT usefulness	0.806	0.873	0.632

Source: own construction using SmartPLS v3.0 (SmartPLS GmbH, Oststeinbek, Germany).

**Table 5:** Outer loadings and outer weights for exogenous variable.

Hypothesis		DT Influence		Innovative CAT Usefulness		Traditional CAT Usefulness	
		**Outer Loading**	**Outer Weights**	**Outer Loading**	**Outer Weights**	**Outer Loading**	**Outer Weights**
H1	SC					0.794	0.325
	**AC**					**0.823**	**0.330**
	PC					0.821	0.326
	MC					0.738	0.274
	ABC			0.856	0.301		
	**TC**			**0.861**	**0.307**		
	LC			0.840	0.280		
	KC			0.815	0.298		
H2	Accessibility	0.787	0.203				
	Customization	0.902	0.226				
	**Rapidity**	**0.953**	**0.248**				
	**Security**	**0.931**	**0.242**				
	Trust	0.823	0.214				

Source: Elaborated by the authors using SmartPLS v3.0.

**Table 6:** Path coefficients.

Hypothesis		Original Sample	Standard Deviation	T Statistics	*p* Values	Validation
H3	DT influence → Innovative CAT improved through DT	0.513	0.040	12.802	0.000	Validated
	DT influence → Traditional CAT improved through DT	0.170	0.033	5.195	0.000	
H4	Innovative CAT improved through DT performance → Improved organizational performance	0.702	0.031	22.641	0.000	Validated
	Traditional CAT improved through DT → Improved organizational performance	0.250	0.031	4.893	0.000	
	Innovative CAT usefulness → Innovative CAT improved through DT	0.466	0.041	11.348	0.000	
	Traditional CAT usefulness → Traditional CAT improved through DT	0.812	0.035	6.703	0.000	

Source: own construction using SmartPLS v3.0 (SmartPLS GmbH, Oststeinbek, Germany).

## Data Availability

Not applicable.

## Abbreviations

AI	Artificial intelligence
BD	Big data
BC	Blockchain
CC	Cloud computing
IoT	Internet of Things
AM	Accounting Management
CAT	Cost Accounting Tools
DT	Digital transformation
SC	Standard Costing
AC	Absorption Costing
PC	Process Costing
MC	Marginal Costing
ABC	Activity-Based Costing
TC	Target Costing
LC	Life Cycle Costing
KC	Kaizen Costing

## Appendix A

**Table A1:** Questionnaire items.

Variables	Items
Demographic variables	What is your gender?
	What is your age range?
Innovative CAT usefulness	On a scale from 1 to 5 (1—not at all useful, 5—very useful), what is the usefulness of ABC for accounting management?
	On a scale from 1 to 5 (1—not at all useful, 5—very useful), what is the usefulness of TC for accounting management?
	On a scale from 1 to 5 (1—not at all useful, 5—very useful), what is the usefulness of LC for accounting management?
	On a scale from 1 to 5 (1—not at all useful, 5—very useful), what is the usefulness of KC for accounting management?
Traditional CAT usefulness	On a scale from 1 to 5 (1—not at all useful, 5—very useful), what is the usefulness of SC for accounting management?
	On a scale from 1 to 5 (1—not at all useful, 5—very useful), what is the usefulness of AC for accounting management?
	On a scale from 1 to 5 (1—not at all useful, 5—very useful), what is the usefulness of PC for accounting management?
	On a scale from 1 to 5 (1—not at all useful, 5—very useful), what is the usefulness of MC for accounting management?
DT influence	On a scale from 1 to 5 (1—not at all important, 5—very important), what do you think is the importance of trust in CAT improved through DT?
	On a scale from 1 to 5 (1—not at all important, 5—very important), what do you think is the importance of security in CAT improved through DT?
	On a scale from 1 to 5 (1—not at all important, 5—very important), what do you think is the importance of rapidity in CAT improved through DT?
	On a scale from 1 to 5 (1—not at all important, 5—very important), what do you think is the importance of customization in CAT improved through DT?
	On a scale from 1 to 5 (1—not at all important, 5—very important), what do you think is the importance of accessibility in CAT improved through DT?
Innovative CAT improved through DT usefulness	On a scale from 1 to 5 (1—not at all useful, 5—very useful), what is the usefulness of ABC improved through DT for accounting management?
	On a scale from 1 to 5 (1—not at all useful, 5—very useful), what is the usefulness of TC improved through DT for accounting management?
	On a scale from 1 to 5 (1—not at all useful, 5—very useful), what is the usefulness of LC improved through DT for accounting management?
	On a scale from 1 to 5 (1—not at all useful, 5—very useful), what is the usefulness of KC improved through DT for accounting management?
Traditional CAT improved through DT usefulness	On a scale from 1 to 5 (1—not at all useful, 5—very useful), what is the usefulness of SC improved through DT for accounting management?
	On a scale from 1 to 5 (1—not at all useful, 5—very useful), what is the usefulness of AC improved through DT for accounting management?
	On a scale from 1 to 5 (1—not at all useful, 5—very useful), what is the usefulness of PC improved through DT for accounting management?
	On a scale from 1 to 5 (1—not at all useful, 5—very useful), what is the usefulness of MC improved through DT for accounting management?
Improved organizational performance	On a scale from 1 to 5 (1—very small, 5—very high), what do you think is the influence of CAT improved through DT on increasing organizational efficiency?
	On a scale from 1 to 5 (1—very small, 5—very high), what do you think is the influence of CAT improved through DT on increasing organizational effectiveness?

Source: own construction based on [67,69,70,71,72].
